# Low genome‐wide divergence between two lizard populations with high adaptive phenotypic differentiation

**DOI:** 10.1002/ece3.8403

**Published:** 2021-12-09

**Authors:** Alejandro Llanos‐Garrido, Javier Pérez‐Tris, José A. Díaz

**Affiliations:** ^1^ Department of Organismic and Evolutionary Biology Harvard University Cambridge Massachusetts USA; ^2^ Department of Biodiversity, Ecology and Evolution UCM Madrid Spain

**Keywords:** genotyping by sequencing, isolation by environment, local adaptation, *Psammodromus algirus*

## Abstract

Usually, adaptive phenotypic differentiation is paralleled by genetic divergence between locally adapted populations. However, adaptation can also happen in a scenario of nonsignificant genetic divergence due to intense gene flow and/or recent differentiation. While this phenomenon is rarely published, findings on incipient ecologically driven divergence or isolation by adaptation are relatively common, which could confound our understanding about the frequency at which they actually occur in nature. Here, we explore genome‐wide traces of divergence between two populations of the lacertid lizard *Psammodromus algirus* separated by a 600 m elevational gradient. These populations seem to be differentially adapted to their environments despite showing low levels of genetic differentiation (according to previously studies of mtDNA and microsatellite data). We performed a search for outliers (i.e., loci subject to selection) trying to identify specific loci with F_ST_ statistics significantly higher than those expected on the basis of overall, genome‐wide estimates of genetic divergence. We find that local phenotypic adaptation (in terms of a wide diversity of characters) was not accompanied by genome‐wide differentiation, even when we maximized the chances of unveiling such differentiation at particular loci with F_ST_‐based outlier detection tests. Instead, our analyses confirmed the lack of genome‐wide differentiation on the basis of more than 70,000 SNPs, which is concordant with a scenario of local adaptation without isolation by environment. Our results add evidence to previous studies in which local adaptation does not lead to any kind of isolation (or early stages of ecological speciation), but maintains phenotypic divergence despite the lack of a differentiated genomic background.

## Graphical abstract

Here, we show that local phenotypic adaptation (supported by a wide diversity of characters and previous experimental work) is not accompanied by genome‐wide differentiation, even when we maximized the chances of unveiling such differentiation at particular loci with FST‐based outlier detection tests. We believe in the importance of adding evidence to previous studies in which local adaptation does not lead to any kind of isolation, as ecologically‐driven divergences seem to be dominant in related bibliography.

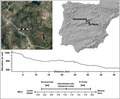

## 1 INTRODUCTION

Overall, the genotypes and phenotypes of populations undergoing local adaptation are expected to be spatially congruent, that is, genetic diversity is expected to follow similar differentiation patterns as adaptive phenotypic diversity (Coop et al., 2010; Friis et al., 2018; Simmonds et al., 2020; Tigano & Friesen, 2016). This positive correlation is commonly known as “isolation by adaptation,” and it is usually studied by estimating ecologically adaptive among‐populations divergence as a proxy of the divergent patterns of selection that cause local adaptation (Funk et al., 2011; Nosil et al., 2008; Schluter, 2000). In theory, isolation by adaptation should be theoretically detectable in the form of divergence peaks among locally selected loci (or among loci genetically correlated with them), but if populations remain isolated long enough, selectively neutral loci can also become differentiated, which makes the detection of outlier loci empirically challenging (Krohn et al., 2019; Llanos‐Garrido et al., 2019a). However, discordances may also appear, whereby two populations may be genetically undifferentiated while showing evident phenotypic divergence (Moody et al., 2015; Palmer & Kronforst, 2015; Shaner et al., 2015). Such discordances can happen, despite the existence of processes that blur overall genomic divergence (e.g., gene flow, recent divergence), only when natural (or sexual; Yang et al., 2018) selection is strong enough to overcome such processes, favoring the presence of locally divergent regions with variants under selection within an otherwise undifferentiated overall genomic background (Burri, 2017; Wang et al., 2019).

While numerous studies have dealt with locally adapted populations occupying different environments or isolated by ecological barriers (Orsini et al., 2013; Rosenblum, 2006; Sexton et al., 2014; Zhao et al., 2020), only a few have focused on the lack of correlation between isolation and adaptation (Feder et al., 2014). Moreover, it has been suggested that there is a significant publication bias against such studies (Krohn et al., 2019). This possible under‐representation of apparent negative results may lead to biased estimates of how frequently local adaptation occurs without isolation, and this is precisely the reason why replicated studies on species or populations with different degrees of isolation are needed (Feder et al., 2014; Sendell‐Price et al., 2020; Talla et al., 2017). In fact, if studies that do not find any effect of environmental gradients on genetic differentiation are rarely published, examples of incipient ecological speciation and/or isolation by environment may artificially be deemed frequent or even widespread (Hendry et al., 2009; Sexton et al., 2014; Shafer & Wolf, 2013).

The aim of this study is to elucidate the patterns of genetic differentiation that underlie phenotypic divergence between two populations of the lacertid lizard *Psammodromus algirus* separated by a 600–700 m altitudinal gradient. This gradient is associated with a large number of habitat differences, including forest type (deciduous vs. perennial) or average annual rainfall (1,170 vs. 438 mm; see the Methods section for a detailed explanation of these habitat differences). The analysis of mitochondrial DNA sequences has shown that these populations present very little genetic differentiation (Díaz et al., 2017; Verdú‐Ricoy et al., 2010), even though they differ in a wide variety of adaptive phenotypic characteristics, including many life history traits (Iraeta et al., 2006, 2011, 2013), sexual ornamentation (Iraeta et al., 2011; Llanos‐Garrido et al., 2017), and prevalence of ticks (Llanos‐Garrido et al., 2017), among others (Table 1). The evidence for such adaptations is based on previous studies that have shown, through reciprocal transplant and common garden experiments, that these adaptive phenotypic differences are not sustained solely by environmental effects (Iraeta et al., 2006, 2013). Therefore, there must be a genetic basis to determine such phenotypic differences between these apparently undifferentiated populations, even if such genetic basis does not lead to an environmentally based isolation. To define the degree of genetic differentiation between the two populations, we used a GBS genomic scan based on 73,291 SNPs that allowed us to analyze the genetic structure and distance between them. In addition, we used a Bayesian method of detection of SNPs with genetic distances between populations greater than expected given the degree of background genomic differentiation (i.e., F_ST_‐based outlier test; Bayescan: Foll and Gaggiotti, 2008). With this approach, we tried to define polymorphisms possibly associated with the patterns of divergent selection that promote the observed adaptive differences (Bonhomme et al., 2010). Thus, if local adaptation has led to isolated populations, we expect to find a genome‐wide differentiation standard that could hamper the detection of divergence peaks at adaptive loci. Alternatively, we might find an undifferentiated genomic background where such peaks should be easy to detect, at least in theory.

**TABLE 1:** Phenotypic differences found between the populations of El Pardo and Navacerrada in previous studies

Phenotypic trait	Reference
Shorter incubation times in the montane population	Iraeta et al. (2006)
Larger hatchlings in the lowland population	Iraeta et al. (2006)
Faster growth in lowland juveniles (reciprocal transplant experiment)	Iraeta et al. (2006)
Faster growth in lowland juveniles (common garden experiment)	Iraeta et al. (2013)
More plastic activity levels in response to food availability in the lowland population	Iraeta et al. (2008)
Larger clutches of smaller eggs in the lowland population	Iraeta et al. (2008, 2013)
Longer flight distance for pregnant females in the montane population	Iraeta et al. (2010)
Relatively longer hind limbs in the lowland population	Iraeta et al. (2011)
Larger adult females in the montane population	Iraeta et al. (2013)
Countergradient variation in body size: the genotypes that presumably control the key adaptations of the lowland population (larger eggs and hatchlings, and faster growing juveniles) occur in a low‐productivity environment in which lizards grow more slowly and reach a smaller adult size	Iraeta et al. (2006), Iraeta et al. (2013)
More and relatively larger femoral pores in males from the lowland population	Iraeta et al. (2011)
Greater development of the sexual coloration of the head (i.e., larger colored surface) in males from the lowland population	Iraeta et al. (2011), Llanos‐Garrido et al. (2017)
Increased saturation of the sexual coloration of the head in males from the montane population	Llanos‐Garrido et al. (2017)
Males respond to the activation of the immune system by reducing the extent of the sexual coloration of the head in the lowland population, and its saturation in the montane population	Llanos‐Garrido et al. (2017)
Higher rates of infestation by tick nymphs in the montane population (no ticks in the lowland population)	Llanos‐Garrido et al. (2017)

We used an approach in which all genetic variants are used to infer the basal level of genomic differentiation, defining outliers as SNPs with a greater degree of divergence and with allelic frequencies deviated from the expected under neutral selection (Lewontin & Krakauer, 1975). The reason why comparing differentiation peaks with adjacent regions should facilitate this task is that the degree of differentiation is heterogeneously distributed throughout the genome (Campagna et al., 2015). Therefore, detecting a divergence peak in a specially conserved region is challenging using delocalized genetic variation, which may find such degree of divergence even below the background genomic differentiation, but which may actually be very divergent within its genomic location (Lawson and Petren, 2017). This scenario is especially common in the coding regions where the genetic basis of phenotypic diversity is located, so that approaches with delocalized SNPs do not usually respond to what is the genetic basis of a given phenotype, but describe the general patterns of genetic differentiation that lay behind the process of local adaptation (e.g., Llanos‐Garrido et al., 2019a; Tigano et al., 2017). Thus, the aim of this study is not to uncover the specific genetic basis behind already published adaptations, but to elucidate whether such local adaptation is accompanied by a significant degree of genome‐wide differentiation or not (Krohn et al., 2019).

## 2 MATERIAL AND METHODS

### 2.1 Study system

*Psammodromus algirus* (Linneus, 1758) is the most abundant lacertid lizard in the Mediterranean scrubland and forest habitats of the Iberian Peninsula (it is only absent from the northern Eurosiberian area). It is a medium‐sized species (snout‐vent length: 60–90 mm; body mass: 6–16 g) in which variation in body size is an important indicator of fitness, as it determines survival, male attractiveness, and female fecundity (Díaz, 1997; Díaz et al., 2005; Iraeta et al., 2013; Martín & Forsman, 1997). *Psammodromus algirus* males present a reddish nuptial coloration during the months of highest activity (April–June) that can range from small sublabial marks in subdominant males to occupy practically the entire head in the largest males. This visual signaling is complemented by the existence of femoral pores as a means of chemical signaling. The number of femoral pores is higher in males and their activity increases as the breeding season advances in response to increased blood androgen levels (Chiu & Maderson, 1975; Cole, 1966; Diaz et al., 1994).

The populations in this study are separated by an altitudinal gradient of 600–700 m and differ in mean annual temperature, precipitation, and habitat structure. Previous studies on these populations revealed very little genetic differentiation inferred with mitochondrial DNA (Verdú‐Ricoy et al., 2010), despite the fact that a wide number of adaptive phenotypic variables are markedly different (Table 1).

The montane population is located at Navacerrada (Cerro de la Golondrina, Sierra de Guadarrama: 40°44′N, 4°00′W; 1,300 m a.s.l.) and its habitat is composed by deciduous oak forests (*Quercus pyrenaica*), scrub patches where *Cistus laurifolius* predominates and, to a lesser extent, granite outcrops and grasslands. The average annual temperature is 6.2°C and the average annual rainfall is 1,170 mm. The lowland population is located at El Pardo (Madrid: 40°31′N, 03°47′W; 650 m a.s.l.), a forest of evergreen holm oaks (*Quercus ilex*) with shrubs of *Cistus ladanifer*. It is separated from the montane population by 32 km in a straight line, and has an average annual temperature of 12.5°C and an average annual rainfall of 438 mm. *Psammodromus algirus* is the most abundant lizard species in both locations, but reaches higher densities at higher altitude (Díaz, 1997).

### 2.2 Field sampling and phenotypic analysis

We noosed or captured by hand 10 males and 10 females from each population (*N* = 40 individuals). Captures were made in the breeding season (April–May) of 2015, and the number of ticks carried by each individual was counted in the field. The lizards were transported to the lab, where all phenotypic traits (snout‐vent length, head width and length, hind leg length, number of femoral pores, and body mass) were measured, and photographs of the throat in ventral position were taken to quantify sexual coloration. For that purpose, we processed the photographs with Adobe Photoshop CS6 as explained by Llanos‐Garrido et al. (2017): we standardized the area of analysis using the “magnetic loop” tool, we measured the red‐colored surface with the “magic wand” tool (with 30% tolerance) after selecting a random red‐colored point, and we then used the “similar” option of the magic wand, with the same tolerance, to select all areas with a similar coloration. The colored surface was measured as the percentage of colored pixels in the area of analysis. To calculate the red saturation, we use the proportion of the red channel within the RGB channel, that is: R/(R + G + B), where R, G and B are the red, green, and blue channels of the graphics card. All these measures were taken blindly with respect to the population of origin. Once all this was done, the lizards were released on their site of capture.

All phenotypic analyses were performed with Statistica software (Statsoft). In order to analyze between populations differences, we used two‐way ANOVAs with sex and population as factors and with different morphological measures as the dependent variables. When necessary, we used ANCOVAs to control for the effect of body size. To assess the relationship between tick load and different explanatory variables (e.g., sex, size, or number of femoral pores), analyses were restricted to the Navacerrada population. To summarize variation in male size in this population, we ran a Principal Component Analysis (PCA) that produced a single factor explaining 84.2% of the variation in the data matrix and giving high factor loadings to all size variables (snout‐vent length, head length and width, mean leg length, and body mass; Table 2).

**TABLE 2:** Principal Component Analysis with morphological variables of males from the Navacerrada population

Feature	Factor loadings
Log Snout‐vent length	0.973
Log Head length	0.928
Log Head width	0.948
Log Hindlimb length	0.769
Log Body mass	0.954
Eigenvalue	4.209
Explained Variance	0.842

Note

All factor loadings have *p*‐values < .001.

### 2.3 DNA extraction and sequencing

We obtained tissue samples by clipping 2 cm of the tail tip of lizards, and we purified DNA for library preparation using the Biotools *Speedtools Tissue DNA Extraction* kit. One of the samples (from the El Pardo lowland population) lacked sufficient DNA to be included in our analyses, leaving a final sample size of 39 genotyped lizards. We used the restriction enzyme *Pst1* to prepare the libraries for genome‐wide genotyping with GBS (*Genotyping by Sequencing*). The sequencing was done in an Illumina HiSeq2500 sequencer, and once the sequences were obtained, we characterized single nucleotide polymorphisms (SNPs). For this purpose, we used the UNEAK pipeline, implemented in TASSEL v.5.0 (Bradbury et al., 2007), and specifically designed for samples of species without a reference genome. The sequences were pruned to eliminate sequencing errors using the error rate threshold parameter. The resulting database consisted of 83,648 SNPs with a coverage of 5.68 ± 6.56 (mean ± SD) and missing site rate of 0.49 ± 0.33. We then discarded loci with minor allele frequencies <0.05, and those that were sequenced in less than 10% of individuals. After this pruning process, the final database consisted of 73,291 loci, with a single SNP per locus and a coverage of 6.6 ± 6.75 and a missingness of 0.42 ± 0.31. This dataset was the one used to analyze the neutral variation of our samples.

### 2.4 Characterization of neutral variation and SNPs under selection

Before performing the genetic structure analysis, we used PLINK v1.9 software (Purcell et al., 2007) to prune the SNPs according to their linkage disequilibrium, estimated by correlation coefficients between SNPs. This filtering step was needed because the subsequent structure analysis does not take into account linkage disequilibrium, and this may lead linked SNPs to bias the grouping of individuals. Genetic structure analyses were performed with the Admixture v.1.3 software (Alexander et al., 2009), which allowed us to estimate the ancestries of each individual through maximum likelihood. To determine the number of groups for which the genetic structure model had more predictive power, we used cross validation errors (cv‐errors). To complement such clustering analysis, we also performed a PCA on TASSEL 5.0 (Bradbury et al., 2007).

We also applied another filtering step before doing the outlier analysis to minimize the false positive rate. We discarded loci whose minor allele frequencies were <0.05 in any population (thus excluding privative alleles) and loci that could not be sequenced in at least 75% of the individuals in each population. The resulting database consisted of 6,421 SNPs. To identify SNPs putatively under selection, we performed an outlier analysis with Bayescan v.2.1. (Foll and Gaggiotti, 2008), a very conservative method which is not prone to false positives, and is very useful when the number of populations is low (Foll and Gaggiotti, 2008). This program uses a logistic regression to split the *F*_ST_ coefficients into a population‐specific effect (*β*) and a locus‐specific effect (*α*). We selected loci with *α* > 0, suggesting positive selection, and a false discovery rate (corrected by multiple testing) of *q* < 0.05.

Once this was done, we tried to annotate SNPs with the highest values of *α*. For this purpose, we run a BLASTn analysis against all the NCBI database. In addition, a Chi^2^ analysis was performed to determine if any of these loci had within population allelic frequencies with significant deviations from what could be expected under Hardy–Weinberg (H‐W) conditions. This was done because the environmental differences described above between the two populations could lead not only to divergent selective pressures in some SNPs, but also to respond to a selective pressure present in one of the populations that is absent in the other (in which case, the deviation of allelic frequencies from what could be expected under H‐W conditions should occur only in that population).

### 2.5 Environmental analysis

**FIGURE 1:**
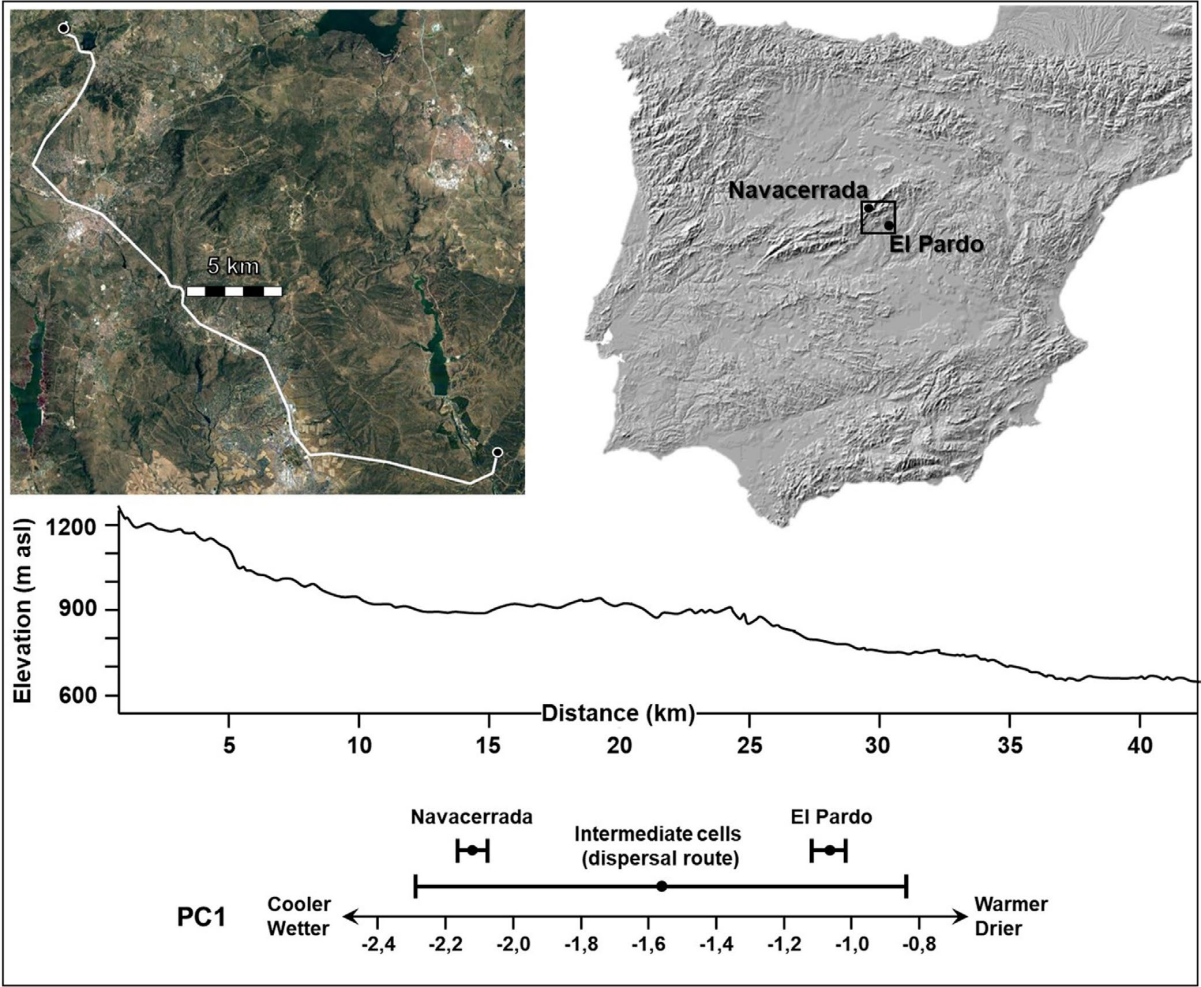
From top to bottom and right to left: location of sampling populations, theoretical straight‐line dispersal route following valley bottoms between them, altitude profile of this route, and the position of both populations and dispersal route (means and SDs) in an environmental gradient described in detail elsewhere (Llanos‐Garrido et al., 2021). These environmental changes are based on an environmental PCA that summarizes BIOCLIM variables on temperature and humidity across all the species' range (annual mean temperature, maximum temperature of the warmest month, mean temperature of the warmest quarter, and annual precipitation)

To evaluate environmental variation between El Pardo and Navacerrada (Figure 1), we computed the score of each cell that an individual would have to cross in a theoretical shortest‐route migration event following valley bottoms on a PCA that combined all Bioclim environmental variables (2.0 dataset; cell resolution = 1 × 1 km; Booth et al., 2014) using R core (R Core Team, 2013). This PCA has been previously used to summarize environmental variation (temperature and humidity) across all the species' range, yielding a single principal axis that opposed dry and warm cells to wet and cold ones (Llanos‐Garrido et al., 2021). To assess the environmental gradient between these populations, we also considered the scores at the cells occupied by the populations themselves.

## 3 RESULTS

### 3.1 Phenotypic variability

**FIGURE 2:**
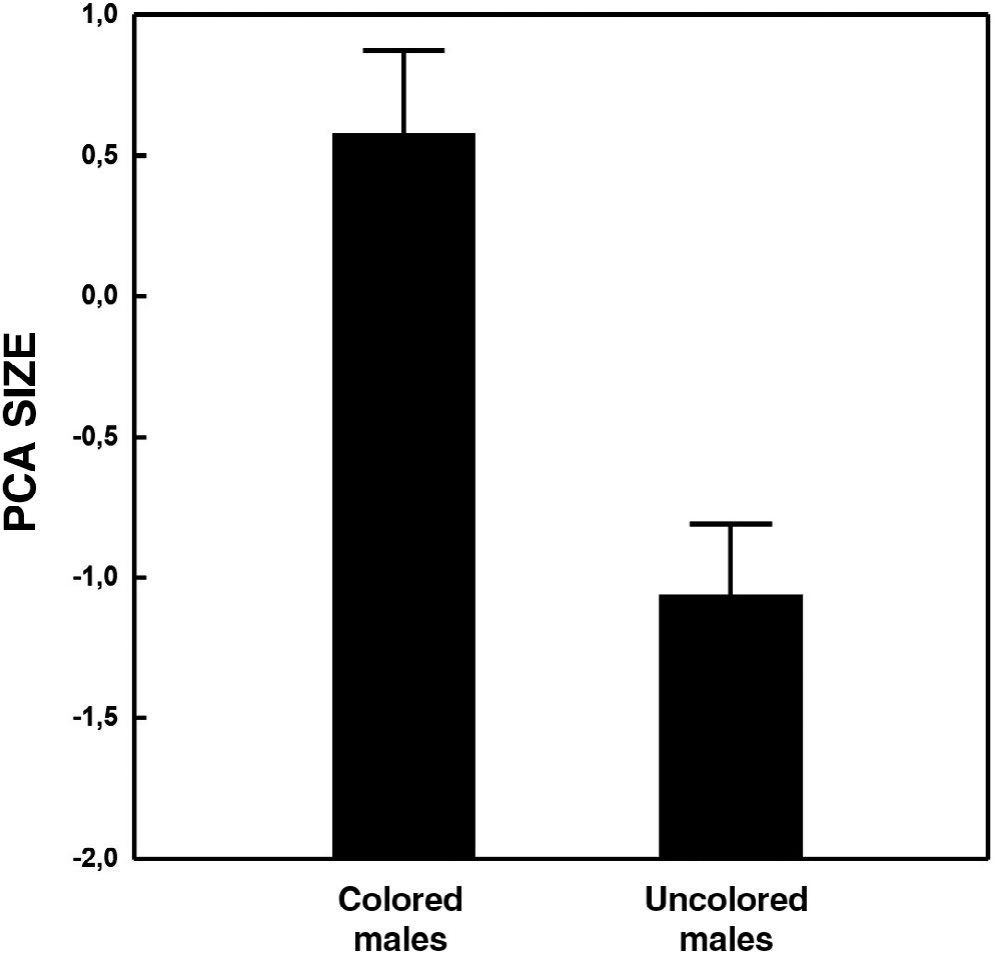
Size differences between males with sexual ornamentation and those without it in Navacerrada (mean ± IC95)

We found significant differences between populations that confirmed the results of previous studies: lizards from the lowland population were smaller and had relatively longer legs than those from the montane population; in addition, they never presented tick infestation, despite adult ticks being present in their habitat (Table 3). Sexual coloration data were especially scarce from males sampled in this study (few colored males with very weak colorations), and as a consequence differences between populations could not be analyzed. When we only considered lizards from Navacerrada, we found that males had more ticks (*F*_1,18_ = 10.85, *p* = .004) and that only the largest males could develop a sexual coloration (*F*_1,8_ = 42.17, *p* < .001; Figure 2). In addition, we detected that, after controlling for the effect of the number of femoral pores (an estimate of each male's investment in chemical signaling) on tick load (*r* = 0.733, *p* < .001), uncolored males tended to have more ticks than colored ones (ANCOVA; number of pores: *F*_1,7_ = 7.54, *p* = .029; color [as factor]: *F*_1,7_ = 3.93, *p* = .088).

**TABLE 3:** Phenotypic differences between the populations studied in this work: results of one‐way ANOVAs with population as factor, and of two‐way ANCOVAs (for the last two variables, marked with an asterisk) with population and sex as factors and size (SVL) as the covariate

Feature	El Pardo (mean ± SD)	Navacerrada (mean ± SD)	Effect of population
Snout‐vent length	68.5 ± 2.5	71.9 ± 5.1	*F*_1,38_ = 6.73; *p* = .01
Head length	10.4 ± 1.2	11.2 ± 1.5	*F*_1,38_ = 3.50; *p* = .07
No. of ticks	0.0 ± 0.0	4.4 ± 4.1	*F*_1,38_ = 66.77; *p* < .001
Hindlimb length*	22.2 ± 0.4	20.6 ± 0.4	*F*_1,35_ = 19.54; *p* < .001
No. of femoral pores*	17.8 ± 0.4	17.1 ± 0.2	*F*_1,35_ = 2.56; *p* = .12

### 3.2 Neutral genetic variability and SNPs under selection

**FIGURE 3:**
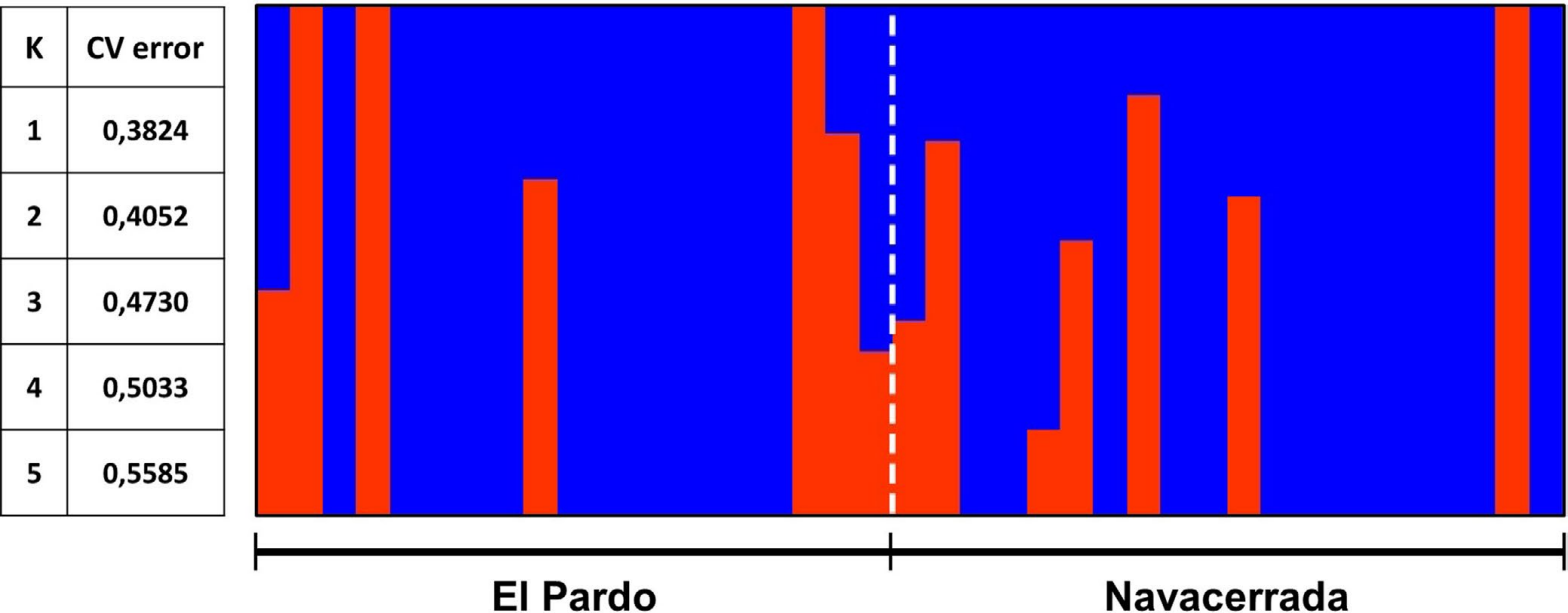
Probability of assignment to groups generated by Admixture for the model with two groups (*K* = 2). On the left, CV‐errors for each of the models analyzed; the most credible model is the one that includes only one group (*K* = 1)

The analysis of genetic structure with admixture yielded a single more plausible model that grouped all samples into a single cluster (*K* = 1, cv‐error = 0.382), indicating no apparent genetic structure between both populations. Even when forcing the model toward higher values of *K* (i.e., less plausible results), we still failed to find any signs of between‐populations genetic structure (*K* = 2, cv‐error = 0.405; Figure 3). A PCA on the covariance matrix obtained from the same data set returned a scree plot that showed a sharp decrease in eigenvalues between PC1 and PC2 (Figure S1), indicating that PC1 was the single axis that best captured overall genetic variation. One‐way analyses of variance showed that both populations had overlapping scores (Figure S2) with means that were nearly identical for PC1 (*F*_1,37_ = 0.000018, *p* = .997) and did not differ significantly for PC2 (*F*_1,37_ = 1.08, *p* = .305).

Regarding outliers analyses, Bayescan did not detect any locus subject to selection (*q*‐values range: 0.883–0.910; mean *F*_st_ ± SD = 0.023 ± 0.0003). However, we explored the loci with largest values of α, the magnitude of the locus‐specific effect of selection, in case they were biologically meaningful. The largest increase in *α* was concentrated on four maximum (although low) values, two of which could not be annotated at all (Table 4). Of the other two, one could be annotated with a marginally nonsignificant *E*‐value of 0.018 (conventional significance value = 0.01), which corresponded to the coding region of a repeated transmembrane ankyrin involved in the cellular response to salicylic acid. The other sequence was annotated with a much higher and nonsignificant *E*‐value (0.6) as the coding region of a nuclear photoreceptor protein. None of these SNPs presented allelic frequencies that deviated significantly from those expected under H‐W conditions after applying the sequential Bonferroni correction (Table 4).

**TABLE 4:** SNPs with greater divergence between populations detected by Bayescan

SNP_ID	*α* (*q*)	H‐W (El Pardo)	H‐W (Navacerrada)
14537818	0.067 (0.883)	*χ* ^2^ = 0.436; *p* = .804	*χ* ^2^ = 0.900; *p* = .638
17216934	0.059 (0.887)	*χ* ^2^ = 5.445; *p* = .066	*χ* ^2^ = 0.183: *p* = .913
17911336	0.050 (0.891)	*χ* ^2^ = 7.871; *p* = .020	*χ* ^2^ = 1.389; *p* = .499
18623045	0.042 (0.885)	*χ* ^2^ = 0.720; *p* = .698	*χ* ^2^ = 0.106; *p* = .949

Note

The Bayescan result (*α* and *q*) and the Chi‐square analysis are presented to verify that the allelic frequencies of these SNPs do not differ significantly from what would expected under Hardy Weinberg's equilibrium.

### 3.3 Environmental variability

By using the scree plot criterion, the environmental PCA yielded a single axis (eigenvalue = 0.679) that retained four variables: annual mean temperature (BIO1), max temperature of the warmest month (BIO5), mean temperature of the warmest quarter (BIO10), and annual precipitation (BIO12) (Llanos‐Garrido et al., 2021). The warmest/driest cells were located near the lowland population (El Pardo), while the opposite was true for the montane population (Navacerrada; see Figure 1). We found significant environmental differentiation between both populations (one‐way ANOVA, *F*_1,38_ = 4802,014, *p*‐value << .0001), as well as relatively high levels of environmental variation along the cells that an individual would have to cross in a theoretical shortest route migration following valley bottoms (mean = −1.563, SD = 0.725; Figure 1). In fact, such variation was much greater than the one found in the geographical cells within any of the two populations, either lowland (mean = −1.069, SD = 0.048; Levene test: *F*_1,28_ = 12.048, *p* = .002) or montane (mean = −2.121, SD = 0.043; Levene test: *F*_1,42_ = 25.807, *p* < .001).

## 4 DISCUSSION

Our results point to a discordance between the absence of genomic divergence and consistent phenotypic differentiation in traits whose adaptive value seems obvious (Iraeta et al., 2006, 2010, 2011, 2013). The fact that, as previously found, in the montane population the load of ectoparasites is related to secondary sexual characteristics of males, and that there are no infestations in the lowland population, shows that the presence of ticks is associated with different selective pressures in both populations (Llanos‐Garrido et al., 2017). Apart from their negative effect on the survival of individuals (Salvador et al., 1996), ticks appear to have other significant impacts on fitness, influencing the expression of sexual ornaments that are important for reproduction (Llanos‐Garrido et al., 2017; Salvador et al., 1996). In addition, the altitudinal gradient involves many other selective pressures that, while less easy to identify than the one imposed by ticks (but still evident given the environmental differences shown by our PCA scores), generate an undeniable impact on fitness‐related traits such as growth rate, egg size, or clutch size (Iraeta et al., 2006, 2010, 2011, 2013). In addition, the adaptive phenotypic differences found in this and earlier studies span the whole life cycle of lizards (e.g., incubation time, hatchling size, juvenile growth rate, and sexual ornamentation and reproductive investment of adults), which should involve a considerable variety of divergent selective pressures underlying their phenotypic differentiation (Roff, 2007). Moreover, the fact that these traits maintain their differentiation in common garden conditions (Iraeta et al., 2013) or in reciprocal transplant experiments (Iraeta et al., 2006) reveals a divergent genetic basis between the two populations (De Kort et al., 2014). However, background genetic differentiation between these two populations is very low (Díaz et al., 2017; Verdú‐Ricoy et al., 2010), even when analytical power is increased with genomic scans. Irrespective of phenotypic differentiation, other populations of *P*. *algirus* showed marked genetic isolation despite being much closer (both geographically and environmentally) than the ones presented here (Díaz et al., 2017; Llanos‐Garrido et al., 2019a). This is true even at a fragmented metapopulation where suitable habitat patches are as close as hundreds of meters apart, and where subpopulations have diverged less than 100 years ago (Pérez‐Tris et al., 2019; Santos et al., 2008). However, this latter scenario occurs at the northern edge of the species' range, where genetic diversity might be lower than at El Pardo or Navacerrada, which occupy the center of the Iberian distribution range (Holt & Keitt, 2000; Takahashi et al., 2016). Such decrease in genetic diversity and/or effective population size could have led to a subsequent reduction of migration rate and increased genetic structure in this marginal population (Deng et al., 2020; Holt & Keitt, 2000; Zalewski et al., 2016), whereas the opposite might be true at the center of the species' distribution range. However, as migration rate between El Pardo and Navacerrada is high enough (or divergence time recent enough) to blur overall genetic structure, it is nearly impossible to properly estimate gene flow among undefined populations (Pfenninger & Posada, 2002). Thus, elucidating how gene flow affects local adaptation in this system constitutes a distinct challenge, really difficult to circumvent even by increasing our analytical power or by getting longer assemblages.

Nevertheless, a low degree of overall genetic structure should not hinder the genetic differentiation of specific variants on which the basis of adaptive divergence is located. There are other systems in which the background genetic differentiation between phenotypically well‐differentiated groups is low, and yet islands of selection whose local divergence outstands above the rest of the genome can still be found (Aguillon et al., 2018). These genome fractions may persist differentiated even in continuous gene flow scenarios between the study groups (Moody et al., 2015; Poelstra et al., 2014; Shaner et al., 2015). In our case, it seems that the divergence between populations is so low that it has not translated into a genomic imprint to a scale large enough to be detected with the approach we have used (Aguillon et al., 2018). However, there is compelling evidence that points to the existence of at least a part of the genome that has diverged, giving rise to the observed phenotypic differences. One possible explanation is that the only divergent regions are those expressed as phenotypes (Safran et al., 2016). These regions would be located in very specific areas of the genome and could be really scarce (Campagna et al., 2015), to the extent that low coverage genotyping approaches could miss them. Another possibility is that the SNPs responsible for the observed adaptive divergence could be located in regions of the genome with low genetic diversity, which would express a peak of divergence at a local genomic scale, but which would escape the outliers detection analysis used in this study (Campbell et al., 2018) if the amount of genetic variability is distributed heterogeneously throughout the genome (Feulner et al., 2015; Nosil et al., 2009; Poelstra et al., 2014). However, such heterogeneity does not seem to be an issue in our system, because background divergence is very low across all the genome, which suggests that SNPs with almost any degree of divergence could have been detected as outliers. Finally, it should be noted that some traits (e.g., size) may have a polygenic basis, in which case the detection of outlier SNPs using Fst‐based approaches could lack enough power to unveil them (De Villemereuil et al., 2014).

Finally, there could be no significant divergence at all on a genome‐wide scale. It is true that previous studies point to a genetic basis, especially those that included reciprocal transplants or common garden experiments, but it could be the case that the observed differences were the result of a heritable epigenetic response to the environment (Groot et al., 2018; Trerotola et al., 2015). Such epigenetic marks could be inherited only up to a specific number of generations, after which the signal would be lost (Trerotola et al., 2015). To discard this hypothesis, we could confirm previous findings by extending common garden experiments a few generations more (Groot et al., 2018), or we could perform a transcriptomic differentiation study (McGirr & Martin, 2018).

In summary, we succeeded to demonstrate that genome‐wide differentiation was not associated with local adaptation in an altitudinal gradient with marked environment differences between two lizard populations. The phenotypic differentiation pattern of these populations was obviously concordant with a scenario of ecologically driven divergence, but it did not lead to overall genotypic isolation between them. Moreover, and for reasons that remain unveiled, genome‐wide differentiation seems much lower than expected by geographic distance. Thus, we uncovered another example of locally (and divergently) adapted populations, which remain undifferentiated on a genome‐wide scale, a phenomenon that could be more frequent than previously thought due to publication bias against this apparent lack of positive results (Hendry et al., 2009). Highlighting natural systems where divergent adaptation occurs with different degrees of genomic‐wide differentiation (including none) is of paramount importance to understand how frequently isolation by adaptation occurs, and to what extent the first incipient stages of ecological speciation are widespread (Feder et al., 2012; Hendry et al., 2009; Krohn et al., 2019; Sexton et al., 2014; Shafer & Wolf, 2013).

## CONFLICT OF INTEREST

None declared.

## AUTHOR CONTRIBUTIONS

**Alejandro Llanos‐Garrido:** Conceptualization (lead); data curation (lead); formal analysis (lead); investigation (lead); methodology (lead); software (lead); writing – original draft (lead). **Javier Pérez‐Tris:** Funding acquisition (lead); project administration (equal); resources (lead); supervision (equal); writing – review and editing (equal). **José A. Díaz:** Formal analysis (supporting); funding acquisition (lead); investigation (equal); project administration (equal); supervision (equal); visualization (lead); writing – review and editing (equal).

## Supporting information

Supplementary Material

## Data Availability

Data for this study are available at PANGAEA (2019b): https://doi.org/10.1594/PANGAEA.908220.
