# Supplementary material for: Low genome‐wide divergence between two lizard populations with high adaptive phenotypic differentiation
Source: Ecol Evol. 2021 Dec 9;11(24):18055–65. doi: 10.1002/ece3.8403 (PMC8717303; doi:10.1002/ece3.8403)

**Supplementary Material**

*Llanos-Garrido et al.* Low genome-wide divergence between two lizard populations with high adaptive phenotypic differentiation

**Figure S1.** Scree plot for the eigenvalues of the five dimensions computed by a Principal Component Analysis on Tassel 5.0 with the 73,291 loci used to analyze variation in our samples.


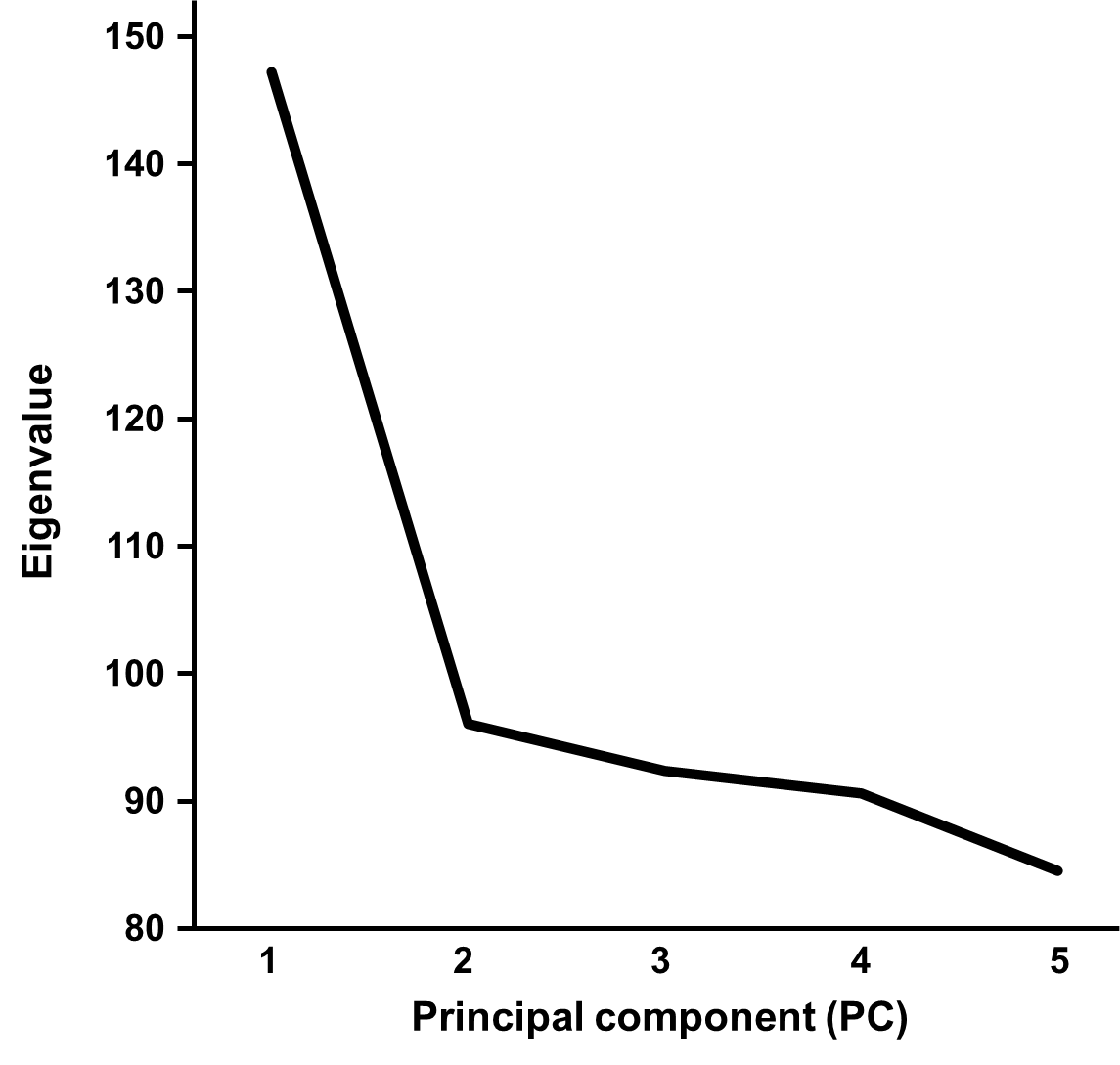


**Figure S2.** Scatterplot of the scores of individual lizards from both populations on PC1 and PC2 of a Principal Component Analysis on genetic variation, showing 95% confidence ellipses for population means. Note the high degree of overlap on both axes, specially on PC1.


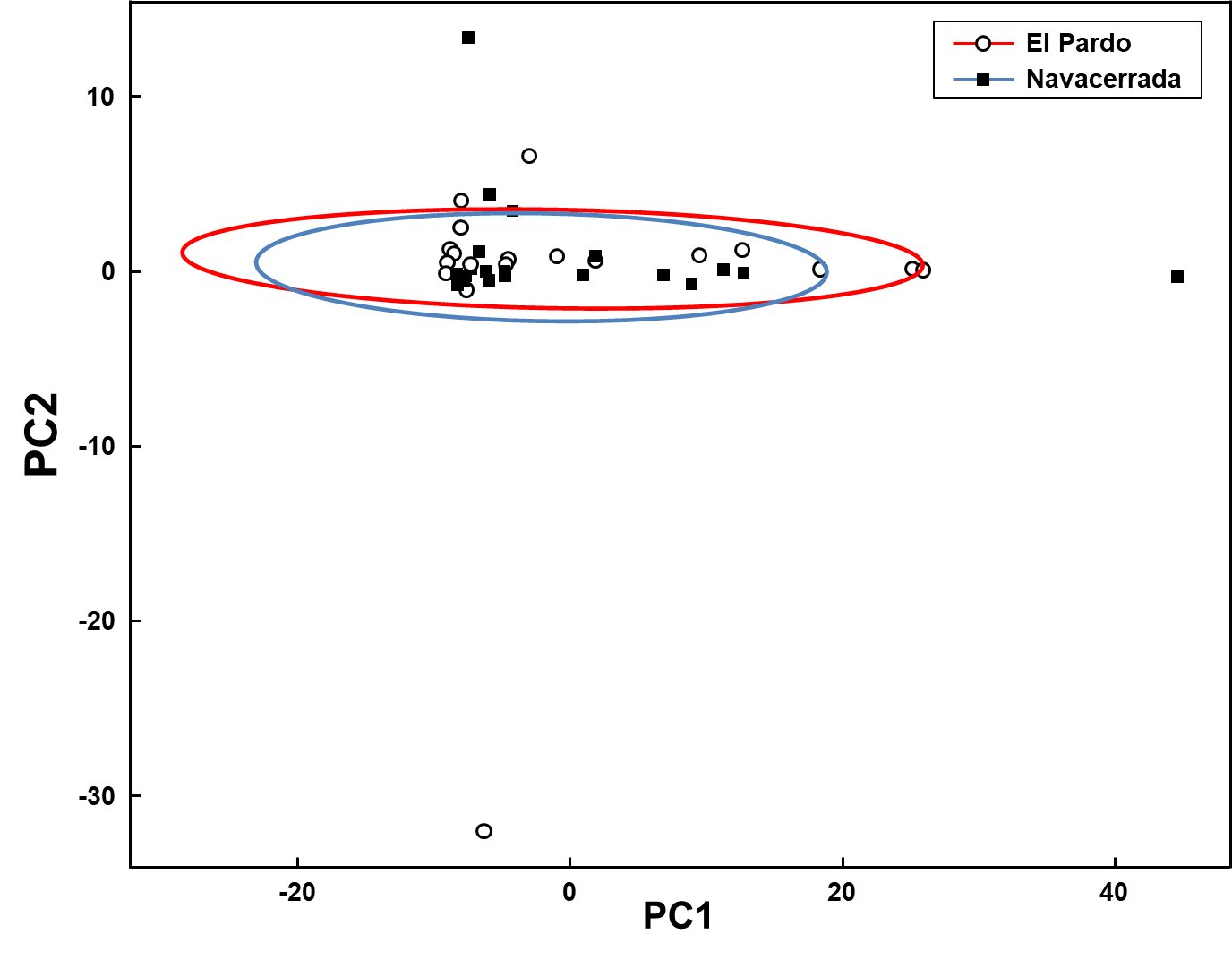

Supplement: Supplementary file 1 — Supplementary Material [file ECE3-11-18055-s001.docx]
